# Anthropometric Characteristics of Triple-Negative Breast Cancer Patients by Menopausal Status: Evidence from the Population-Based Multicentric Study—MCC-Spain

**DOI:** 10.3390/healthcare14030321

**Published:** 2026-01-27

**Authors:** Marina Muñoz-Pérez, Lorena Botella-Juan, Facundo Vitelli-Storelli, Virginia Lope, Mireia Obón-Santacana, Pilar Amiano, Marcela Guevara, Guillermo Fernández-Tardón, Juan Alguacil, Sonia del Barco, Ana Molina-Barceló, Trinidad Dierssen-Sotos, Antonio José Molina, Vicente Martín-Sánchez, Gemma Castaño-Vinyals, Beatriz Pérez-Gómez, Manolis Kogevinas, Marina Pollán, María Rubín-García

**Affiliations:** 1 Department of Biomedical Sciences, Area of Preventive Medicine and Public Health, Faculty of Health Sciences, University of León, 24071 León, Spain; mmunop00@estudiantes.unileon.es (M.M.-P.); ajmolt@unileon.es (A.J.M.); vmars@unileon.es (V.M.-S.); mrubig@unileon.es (M.R.-G.); 2 Group of Investigation in Interactions Gene-Environment and Health (GIIGAS), Institute of Biomedicine (IBIOMED), University of León, 24071 León, Spain; vitelli@uji.es; 3 Consortium for Biomedical Research in Epidemiology & Amp, Public Health, CIBERESP, Carlos III Institute of Health, 28029 Madrid, Spaintrinidad.dierssen@unican.es (T.D.-S.);; 4 Perinatal Epidemiology, Environmental Health and Clinical Research, School of Medicine, Universitat Jaume I, 12071 Castellon, Spain; 5 Cancer and Environmental Epidemiology Unit, Department of Epidemiology of Chronic Diseases, National Center for Epidemiology, Instituto de Salud Carlos III (ISCIII), 28029 Madrid, Spain; 6 Unit of Biomarkers and Susceptibility (UBS), Oncology Data Analytics Program (ODAP), Catalan Institute of Oncology (ICO), L’Hospitalet del Llobregat, 08908 Barcelona, Spain; 7 ONCOBELL Program, Bellvitge Biomedical Research Institute (IDIBELL), L’Hospitalet de Llobregat, 08908 Barcelona, Spain; 8 Public Health Division of Gipuzkoa, Biogipuzkoa Research Institute, Av. Navarra 4, 20013 San Sebastian, Spain; 9 Instituto de Salud Pública y Laboral de Navarra (ISPLN), IdiSNA, 31003 Pamplona, Spain; 10 Health Research Institute of Asturias (ISPA), Av. Hospital Universitario s/n, 33011 Oviedo, Spain; 11 Centro de Investigacion en Recursos Naturales, Salud y Medio Ambiente (RENSMA), Universidad de Huelva, 21071 Huelva, Spain; 12 Descriptive Epidemiology, Genetic and Cancer Prevention Group, Institut d’ Investigació Biomèdica de Girona Dr. Josep Trueta (IDIBGI-CERCA), 17005 Girona, Spain; 13 Medical Oncology, Catalan Institute Oncology (ICO), 17007 Girona, Spain; 14 Cancer and Public Health Research Unit, Foundation for the Promotion of Health and Biomedical Research of Valencia Region (FISABIO-Public Health), 46020 Valencia, Spain; ana.molina@fisabio.es; 15 Department of Medical and Surgical Sciences, Faculty of Medicine, University of Cantabria, 39011 Santander, Spain; 16 Valdecilla Health Research Institute (IDIVAL), 39011 Santander, Spain; 17 Barcelona Institute for Global Health (ISGlobal), 08036 Barcelona, Spain; 18 Department of Experimental and Health Sciences, Universitat Pompeu Fabra (UPF), 08003 Barcelona, Spain; 19 Hospital del Mar Medical Research Institute, 08003 Barcelona, Spain

**Keywords:** triple-negative breast cancer, menopausal status, body composition

## Abstract

**Background/Objectives**: This study aimed to analyze the relationship between various anthropometric measurements (Body Mass Index (BMI), Clínica Universidad de Navarra-Body Adiposity Estimator (CUNBAE), hip and waist circumference (WC), weight, and height) and Triple-Negative Breast Cancer (TNBC) according to menopausal status. **Methods:** A total of 113 TNBC cases and 226 matched controls from the MCC-Spain study were included. Controls were matched by age, educational level, family history, and province. Conditional logistic regression models, stratified by menopausal status, were used to estimate adjusted Odds Ratios (aORs) and their 95% Confidence Intervals (95% CIs) for the association between anthropometric measures and TNBC risk. **Results**: A divergent non-significant trend was observed: compared to their respective controls, premenopausal cases tended to have lower mean anthropometric measurements (except height), while postmenopausal cases showed higher means. No statistically significant associations were observed for individual measures derived from logistic regressions. However, when comparing women with normal BMI and normal WC (the reference group), a non-significant association of risk was found in those premenopausal women who were centrally obese (normal weight/high WC) (aOR = 1.79; 95% CI = 0.17–18.29), but the combination of overweight and a large WC showed an aOR of 0.22 (95% CI = 0.03–1.68) before menopause. In contrast, the combination of overweight and a high WC showed a statistically significant adjusted OR of 3.28 in postmenopausal women (95% CI = 1.10–9.81). **Conclusions:** Our findings suggest that the relationship between adiposity and TNBC is inverse in premenopausal women and direct in postmenopausal women, highlighting the importance of considering both body fat distribution and menopausal status when evaluating TNBC. However, our findings are limited by low statistical power, which may have led to a lack of statistical significance, and there is a need for larger, collaborative studies.

## 1. Introduction

Worldwide, one in every four cancers affecting women is breast cancer. With more than 2.2 million new cases annually and nearly 700,000 deaths, it is one of the most significant public health challenges globally [1]. In the Spanish context, it is the tumour with the highest incidence (representing 30.3% of all incident cases) and was the leading cause of cancer death (14.7% of all cancer deaths) in women in 2022 [2].

Breast cancer is typically classified into subtypes based on the presence of estrogen and/or progesterone hormone receptors (HR+) and HER2 receptors. The immunohistochemical absence of all three receptors is known as Triple-Negative Breast Cancer (TNBC). This molecular subtype accounts for 10–20% of cases. It is resistant to hormonal and HER2-directed therapies and is often associated with higher case fatality rates and increased risk of distant metastases due to its high genomic instability, high mitotic index, and general heterogeneity [3,4,5].

Of the modifiable risk and lifestyle factors that influence breast cancer, obesity is one of the most significant due to its multiple mechanisms of action. The relationship between obesity and breast cancer varies significantly according to a woman’s menopausal status. The World Cancer Research Fund states that, while excess body fat is an established risk factor for breast cancer in postmenopausal women, it is a probable protective factor in premenopausal women [6].

However, the available evidence on excess fat and TNBC is limited and inconclusive. While some studies suggest that excess body fat may be related to an increased risk of TNBC and a poorer prognosis in premenopausal women, the evidence for this relationship is often unclear in postmenopausal women. It has frequently shown no effect [7,8,9,10].

One of the main explanations for the difference in behavior between cancer subtypes lies in the variable exposure to estrogen mediated by body fat [11]. Unlike other subtypes, the association between obesity and TNBC is predominantly mediated by non-hormonal pathways, such as chronic inflammation and hyperinsulinemia, rather than by estrogen production. Specifically, adipocytes cause chronic subclinical inflammation, leading to increased systemic levels of cytokines (IL-6 and TNF-α) that stimulate tumor cell proliferation, invasion, and resistance to treatment [7]. Obesity also induces hyperinsulinemia, which promotes tumor growth independently of hormone receptors [11].

Reliance on these non-hormonal pathways highlights the need to go beyond standard indicators, such as the body mass index (BMI), when studying the impact of body fat on TNBC development. Other anthropometric measures provide a more comprehensive evaluation of nutritional status and body fat distribution; factors that may influence the development of specific breast cancer subtypes, such as TNBC [12]. These measures include weight, height, waist circumference (WC), and hip circumference (HC), which estimate central adiposity and high metabolic risk. Additionally, a relevant tool is the CUNBAE (Clínica Universidad de Navarra-Body Adiposity Estimator), which provides a more accurate approximation of the distribution of people’s body fatness profile [13].

Given the aggressiveness and severity of TNBC, the complex, non-hormonal mechanisms linking it to adiposity, and the inconsistent evidence regarding menopausal status, a deeper understanding of this association is necessary to identify those at highest risk and to develop effective prevention strategies.

Therefore, the objective of this study was to analyze the relationship between different anthropometric measurements (including BMI, CUNBAE, weight, height, WC, and HC) and TNBC, specifically examining how this association differs between premenopausal and postmenopausal women.

## 2. Materials and Methods

### 2.1. Participants

The participants were part of the Multicase-Control study (MCC-Spain), a multicentre case–control study conducted in 23 hospitals across 12 Spanish provinces. Its objective was to evaluate environmental exposures and their interactions with genetic factors for a variety of tumors, including breast cancer [14,15].

MCC-Spain cases were identified through an active search that included periodical visits to the collaborating hospital departments. All included cases were incident with histological confirmation, diagnosed between 2008 and 2013. They resided in the hospitals’ recruitment areas for at least 6 months before recruitment. Only women who signed an informed consent form were recruited; the informed consent form also requested their permission to subsequently consult their medical records during follow-up. Controls were selected from the general population according to the age and sex distribution of the cases included in the study.

For the present analysis, a subsample of MCC-Spain (N = 339) was selected, consisting of the women with histological confirmation of TNBC (n = 113 cases). The 226 controls were selected from the total number of controls in the study and matched to the cases at a 2:1 ratio using the Propensity Score Matching (PSM) tool in R with the nearest neighbour method. The matching covariates used in the PSM included age, educational level, first-degree family history of breast cancer, and area of residence. The quality of the matching was verified so that the case and control groups had balanced covariates with similar distributions. The standardised differences in means between the two groups for the main variables did not exceed the ±0.1 quality threshold.

The MCC-Spain protocol was approved by the ethics committees of the participating institutions. Information on ethics and data availability is offered at https://www.mccspain.org. Data confidentiality was ensured by removing personal identifiers from the datasets. The database was registered with the Spanish Data Protection Agency under number 2102672171.

### 2.2. Study Variables

Information was collected using a structured, computerized questionnaire. Trained interviewers administered this in a face-to-face interview lasting approximately 60 min, during which participants provided self-reported information on various factors. From the data available, we considered the following for this study:

Sociodemographic data: age (years), educational level (categorized as below primary, primary, secondary, and university), and province of reference hospital (León, Barcelona, Madrid, Asturias, Cantabria, Gipuzkoa, Navarra, Granada, Huelva, Murcia, and Valencia).

The following self-reported anthropometric data were used for the analyses: weight (kg), height (cm), WC (cm), and HC (cm). BMI was calculated from these measurements (kg/m^2^). In addition, CUNBAE was calculated using the variables BMI, sex and age, and the result is expressed as a percentage of body fatness [13].

Other variables were used, such as history of breast cancer in first-degree relatives, menopausal status, age at menarche (≤12 years or >12 years), alcohol consumption (0, 0–12 or ≥12 g/day), physical activity level (≤8 or >8 MET×h/week), and nulliparity (yes or no). When a value was missing, it was treated as an additional category and included in the multivariate analysis to avoid losing sample size.

### 2.3. Data Analysis

The descriptive characteristics of the sample were analyzed using measures of central tendency and dispersion (mean and standard deviation (SD)) for quantitative variables and absolute and relative frequencies for qualitative variables. A descriptive analysis of the cases and controls included was performed, considering the variables age, area of residence, level of education, family history of breast cancer, menopausal status, age at menarche, nulliparity, alcohol consumption, physical activity level, and anthropometric measurements.

For the graphical representation of anthropometric measurements, variables were normalized using the control group values for each menopausal status category as a reference. Normalization was performed by calculating Z-scores (the ratio of each value’s deviation from the mean to the SD) to standardize units of measurement and enable appropriate comparisons.

Student’s *t*-test was used to evaluate differences in the means of the anthropometric variables between cases and controls.

Multivariate conditional logistic regression models were used to analyze the possible association between anthropometric measurements and the risk of TNBC, yielding odds ratios (ORs) and their respective 95% confidence intervals (95% CIs). All anthropometric variables were assessed separately and then BMI and WC were combined to create four categories, according to WHO criteria combined recommendations cut-off points [16]: Normal weight (<25 kg/m^2^)/Normal WC (≤88 cm), Normal weight (<25 kg/m^2^)/High WC (>88 cm), Overweight (≥25 kg/m^2^)/Normal WC (≤88 cm), and ≥Overweight (≥25 kg/m^2^)/High WC (>88 cm).

A first unadjusted model was carried out (model 1—matched), and a second model (model 2—fully adjusted) adjusted made for age at menarche (≤12 years or >12 years), nulliparity (yes or no), alcohol consumption (0, 0–12 or ≥12 g/day), and physical activity level (≤8 or >8 MET×h/week).

Logistic linear models were compared with restricted cubic splines to assess linearity between anthropometric measurements and the risk of TNBC. All statistical analyses were performed using STATA 19.0 [17] and R v.024.09.0.

## 3. Results

Table 1 shows the characteristics of the 113 TNBC cases and their 226 matched controls. The mean age of the sample analyzed was 57.0 (12.7) years for controls and 55.9 (13.9) years for cases. There was a higher proportion of postmenopausal women (66.0%) compared to premenopausal women (34.0%). The similar distribution of cases and controls across age, province, educational level, and family history of breast cancer verified the good matching of controls to cases.

**Figure 1:**
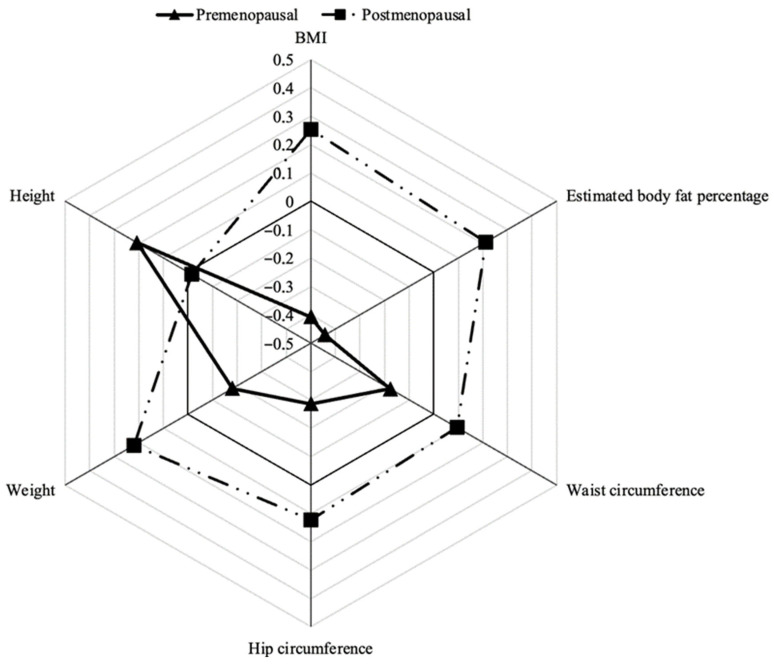
Standardised differences in anthropometric measurements between cases and controls according to menopausal status. Abbreviations: BMI = body mass index.

Table 2 details the anthropometric characteristics of cases and controls in the total sample and stratified by menopausal status. No statistically significant differences were observed between cases and controls, either overall or within groups by menopausal status. However, when the data were stratified among premenopausal women, TNBC cases tended to show lower values for all measures than controls. In contrast, the opposite pattern occurs in the group of premenopausal women; cases tended to show slightly higher values across all the measurements than their controls. This divergent pattern based on the stage of menopause becomes clearly evident when comparing the Z-scores of the cases with those of the controls (Figure 1). Except for height, postmenopausal women with TNBC showed higher Z-values in anthropometric measurements than their controls, suggesting a tendency towards increased overall and central adiposity in this case group. The opposite pattern occurred in the premenopausal group; women with TNBC exhibited lower Z-values in most measurements compared to their controls, although they were slightly taller.

Table 3 shows the association between anthropometric indicators and TNBC in the total sample of women and stratified according to menopausal status. To assess whether the results from the logistic regressions were appropriate, the model linearity was verified. No differences (*p* > 0.05) were found between the linear logistic models with restricted cubic splines used to test linearity, and no relationship other than linear was observed.

According to Table 3, in the total sample, none of the continuous measures (BMI, CUNBAE, WC, HC, weight, or height) showed statistically significant associations with TNBC in either the matched (Model 1) or the fully adjusted model (Model 2). All ORs were close to 1, and their 95% CI included the null value. Regarding the combined BMI–WC categories, none of the adiposity patterns differed significantly from the reference group (Normal weight/Normal WC). Although all categories (compared with the reference group) showed slightly higher odds of TNBC, the association did not reach statistical significance in any model.

At the same time, in Table 3, the results of conditional logistic regression according to menopausal status are shown. In contrast with the results found in the whole sample, according to menopausal status in the group of premenopausal women, BMI, CUNBAE, WC, HC, and weight were associated with a non-significant protective trend with TNBC. While in postmenopausal women these anthropometric measures tended to act as risk factors, in particular, body weight showed a significant positive association with TNBC risk (aOR = 1.05; 95% CI 1.01–1.09).

Considering BMI and WC as categorical variables, in premenopausal women, there is a protective trend (non-statistically significant) for higher BMI and WC with TNBC (OR = 0.23). In contrast, in postmenopausal women, there is a risk trend, with ORs greater than 2 in the higher BMI and WC categories (fully adjusted model 2). When BMI and WC were analyzed jointly, we observed that any combination, compared with being of normal weight and having a normal WC, tended to increase the risk of TNBC in postmenopausal women. This risk reached statistical significance specifically for women classified as ≥Overweight/High WC (simultaneous overall and central adiposity) compared to the reference group (Normal weight/Normal WC) (aOR = 3.28; 95% CI 1.10–9.81). In the case of premenopausal women, a non-significant association of risk was found in those women who were centrally obese (normal weight/elevated WC) (aOR = 1.79; 95% CI = 0.17–18.29). However, the simultaneous presence of central and abdominal obesity behaves as a protective factor, although it does not reach statistical significance in the fully adjusted model (Model 2; adjusted OR = 0.22, 95% CI = 0.03–1.68).

## 4. Discussion

The results of analyzing the relationship between anthropometric measurements and TNBC showed that, when menopausal status was not stratified, there were no differences in the means of the anthropometric measurements between the case and control groups. However, when the results were stratified by menopausal status, some differences in trends were observed, although they were not statistically significant. Premenopausal cases showed lower anthropometric measurement means than their matched controls, while postmenopausal cases showed higher anthropometric values than their controls, except for height. In the case of height, however, the opposite pattern was observed, with a negative correlation between higher values and TNBC in postmenopausal women.

According to the multivariate conditional logistic regression models, no significant associations were found between any anthropometric measures and risk of TNBC in the whole sample. No statistically significant differences were found in any continuous measure when considering menopausal status. However, a protective trend was observed for higher anthropometric measurements in premenopausal women, and a slightly increased risk was observed in postmenopausal women. Although these results should be treated with caution due to the small sample size, this finding contradicts existing evidence for premenopausal women, for whom obesity has been associated with an increased risk of TNBC [9,18]. But this result is in line with some articles about postmenopausal women where obesity appears as a non-conclusive factor for the TNBC [19,20].

Interestingly, the combined analysis of BMI and abdominal fat distribution (measured by WC) yielded some results, although the 95% CI were extensive due to categorisation. While BMI or WC individually appeared to be possible protective factors for TNBC in premenopausal women, it was observed that those who were of normal weight but had a large WC had a higher risk when both factors were considered together. This may be due to fat distribution, suggesting that central adiposity and visceral fat could increase TNBC risk. However, in this group of women, having a large WC and obesity emerged as a protective factor. This finding lends weight to the idea that the distribution of body fat may be a relevant factor in the development of TNBC in premenopausal women [21], a fact already discussed in the recent literature [21].

These results are convergent with other studies that found that the risk of TNBC was reduced for women with a high BMI but elevated for those with a body fat distribution pattern compatible with central obesity [22], and with a recent study about the relationship of abdominal and visceral fat and an increased risk of TNBC in all cases [23]. Furthermore, according to His et al. [24] certain patterns of fat distribution, such as central and gluteo-femoral adiposity, are positively associated with the risk of this type of cancer, observing a direct relationship between WC and breast cancer risk in premenopausal women, even after adjusting for BMI and in women of normal weight. These findings support the possibility that TNBC tumours may be more influenced by a sum of components and other phenomena such as metabolic syndrome [25].

In postmenopausal women in our study, we observed that higher BMI and WC, as well as the combination of all weight and body fat distribution patterns (WC), significantly increase the risk of TNBC, especially in women with obesity and a large WC. This contradicts the results of several authors, who found no clear effect as indicated above. However, other studies supported the idea that central adiposity in these women is a key factor in the increased risk of TNBC development [26]. In this regard, Van Mieghem T. [27] proposes a possible pathophysiological explanation: the increase in estrogen levels associated with postmenopausal obesity could reduce HER2 receptor expression, thus favouring the development of the TNBC subtype.

As with premenopausal women, interpreting the associations between different measures of adiposity remains complex, which reinforces the idea that the relationship between adiposity and TNBC may be influenced by multiple factors [28,29]. Strong correlations between WC and elevated insulin levels may help explain the relationship between a larger WC and an increased risk of breast cancer. Similarly, characteristics of gluteal-femoral adipose tissue, of which WC is a marker, such as its hormone secretion profile, may contribute to this association [24]. Furthermore, it has been suggested that obesity in postmenopausal women may induce chronic inflammation characterised by increased cytokines and insulin resistance, which could promote tumour development [30].

It should be noted that the literature has significant methodological limitations, particularly in studies focusing on young women with TNBC. These limitations are mainly due to the small sample size, given that TNBC is the least common cancer subtype. Therefore, we reiterate that studying each anthropometric measurement individually may have a low impact on TNBC development. We believe that combined measurements could be a better indicator of TNBC development than weight and fat distribution alone.

### Limitations and Strengths

This study has several limitations that should be considered when interpreting the results. First, the sample size. Given that TNBC is a less frequent subtype within breast neoplasms, the analysed sample was relatively small, which may have limited the statistical power to detect significant associations, especially in analyses stratified by menopausal status. Furthermore, estimates may have been inaccurate for this same reason, as confidence intervals were very wide. Also, the inherent limitations of a case–control study should be noted. These limitations include selection and recall bias, the case–control design prevented us from establishing causality. Therefore, we must be cautious in interpreting these results. Additionally, another limitation of the design is that the anthropometric measures were self-reported, which may have led to underestimation of weight and overestimation of height. However, this measurement report was the same in cases as in controls, so it is considered a non-differential error that allows comparability.

Despite these limitations, the study also has significant strengths. Among these, the exclusive inclusion of histologically confirmed incident cases of TNBC stands out, which ensuring diagnostic validity and allows for a more specific approach to this tumour subtype, which has distinct biological and prognostic characteristics. As for the selection of controls, using lists of general practitioners provided a population-based sample, given the universal public coverage of the National Health System in Spain, which is another strength of the study. In addition, for this substudy, controls were matched to TNBC cases by age, educational level, first-degree family history, and province of residence, ensuring that both groups were comparable, assessing the good quality of the matching. Another major strength is the use of data from the MCC-Spain project, a large, well-established, multicentric study with rigorous methodological and ethical standards. Likewise, the sample covers multiple geographical regions of Spain, contributing to greater heterogeneity among participants in terms of educational level, anthropometric distribution, and other sociodemographic factors. This diversity improves the applicability of the results, as it allows the study’s conclusions to be more representative of the Spanish female population in general.

This study focuses on TNBC, which has the poorest prognosis and is underrepresented in the literature due to its low prevalence. In this study, we also considered menopausal status, which is a key factor in breast cancer. This contributes to the study’s novelty and originality. Addressing this gap provides novel evidence in an area with limited data, helping to mitigate publication bias and bringing results that would otherwise be overlooked due to limited statistical significance to the attention of the research community. It is also one of the few epidemiological articles in the literature to focus specifically on the TNBC subtype, treating it as the primary focus rather than just another outcome. Crucially, making these data publicly available is intended to encourage other researchers to utilise and share existing TNBC datasets, thereby facilitating pooled analyses and enhancing statistical power. Finally, this study’s focus on the relationship between different anthropometric measurements and TNBC specifically is valuable.

## 5. Conclusions

No significant associations were observed between individual anthropometric measures and TNBC risk in the overall sample. However, stratification by menopausal status revealed distinct patterns. In premenopausal women, higher anthropometric values showed a possible protective trend, while central adiposity in women with normal BMI showed estimates consistent with an increased risk. In postmenopausal women, a higher BMI and waist circumference, particularly when combined, showed estimates consistent with an increased risk of TNBC. Therefore, the distribution of body fat appears to have the most significant impact. Nevertheless, further research into TNBC is required, given that existing studies have a small number of cases. This study provides epidemiological evidence focusing on TNBC, the breast cancer subtype with the poorest prognosis, incorporating menopausal status as a key analytical dimension. The availability of these data aims to facilitate pooled analyses with other researchers and improve understanding of the relationship between anthropometric measures and TNBC, paving the way for further research into this phenomenon.

## Figures and Tables

**Table 1:** Descriptive characteristics of the sample according to cases and controls.

	Controls TNBC (n = 226) 66.67%		Cases TNBC (n = 113) 33.33%	
	n/M	%/SD	n/M	%/SD
Age (years)	57.0	12.7	55.9	13.9
Area				
Asturias	18	8.0	6	5.3
Barcelona	46	20.4	22	19.5
Cantabria	1	0.4	2	1.7
Girona	8	3.5	4	3.5
Guipúzcoa	25	11.1	13	11.5
Huelva	9	4.0	5	4.4
León	49	21.7	23	20.4
Madrid	48	21.2	23	20.4
Navarra	17	7.5	11	9.7
Valencia	5	2.2	4	3.5
Level of education				
Less than primary school	30	13.3	14	12.4
Primary education	80	35.4	41	36.3
Secondary education	81	35.8	40	35.4
University	35	15.5	18	15.9
Family history of breast cancer				
Yes	24	10.6	9	8.0
No	202	89.4	104	92.0
Menopausal status				
Premenopausal	75	33.2	38	33.6
Postmenopausal	151	66.8	75	66.4
Age at menarche (years)				
≤12	105	46.5	46	40.7
>12	120	53.1	65	57.5
Missing	1	0.5	2	1.8
Nulliparity				
Yes	39	17.3	18	83.2
No	186	82.3	94	15.9
Alcohol consumption (g/day)				
0	57	25.2	30	26.3
0–12	117	51.8	50	44.3
≥12	25	11.1	9	8.0
Missing	27	12.0	24	21.2
Physical activity level (MET×h/week)				
≤8	114	60.64	66	71.0
>8	71	37.77	27	29.0
Missing	3	1.60	0	0.00

Abbreviations: M = mean, SD = standard deviation. TNBC = Triple-negative Breast Cancer.

**Table 2:** Anthropometric measurements of triple-negative breast cancer cases and controls of the total sample and by menopausal status.

	Total					Premenopause					Postmenopause				
Anthropometric Measures	Cases		Controls		*p*-Value *	Cases		Controls		*p*-Value *	Cases		Controls		*p*-Value *
	Mean	SD	Mean	SD		Mean	SD	Mean	SD		Mean	SD	Mean	SD	
BMI (kg/m^2^)	26.1	5.0	25.9	4.7	0.8	23.7	3.1	25.1	5.0	0.1	27.3	5.3	26.3	4.6	0.2
CUNBAE (%)	38.4	6.4	38.4	6.2	0.7	33.6	4.7	35.6	6.6	0.1	40.8	5.8	39.8	5.4	0.2
Waist circumference (cm)	103.4	9.5	104.0	10.6	0.8	98.6	8.1	101.6	10.6	0.1	105.4	11.0	104.3	8.8	0.4
Hip circumference (cm)	88.1	13.3	88.1	13.9	1.0	82.0	10.3	84.5	14.2	0.3	91.2	13.6	89.9	13.5	0.5
Weight (kg)	65.5	11.5	66.2	12.3	0.6	62.5	9.2	65.0	12.9	0.3	68.1	13.3	65.7	10.9	0.2
Height (cm)	159.2	6.4	159.6	6.8	0.6	162.3	5.5	161.0	6.5	0.3	158.2	7.0	158.3	6.2	0.9

* *p*-value indicates the results obtained for the *t*-test comparing means. Abbreviations: BMI: Body Mass Index; CUNBAE: University of Navarra Clinic—Body Fat Estimator; SD = standard deviation.

**Table 3:** Association between anthropometric measurements and triple-negative breast cancer in the total sample and premenopausal and postmenopausal women using conditional logistic regression models.

	Model 1—Matched ^a^						Model 2—Fully Adjusted ^b^					
	Total		Premenopause		Postmenopause		Total		Premenopause		Postmenopause	
	OR	CI 95%	OR	CI 95%	OR	CI 95%	OR	CI 95%	aOR	CI 95%	aOR	CI 95%
Continue measures												
BMI (per 1 kg/m^2^)	1.01	0.96–1.06	0.92	0.78–1.07	1.03	0.97–1.09	1.04	0.98–1.10	0.83	0.64–1.08	1.02	0.96–1.09
CUNBAE (per 1%)	1.00	0.96–1.04	0.94	0.86–1.04	1.03	0.97–1.08	1.02	0.97–1.07	0.88	0.74–1.04	1.02	0.96–1.08
WAIST CIRCUMFERENCE (per 1 cm)	1.00	0.98–1.02	0.97	0.92–1.02	1.01	0.98–1.03	1.01	0.99–1.03	0.97	0.92–1.03	1.03	0.10–1.07
HIP CIRCUMFERENCE (per 1 cm)	1.00	0.97–1.02	0.97	0.91–1.02	1.01	0.98–1.04	1.01	0.98–1.04	0.97	0.91–1.03	1.03	0.99–1.08
WEIGHT (per 1 kg)	1.01	0.99–1.02	0.98	0.94–1.03	1.02	0.99–1.05	1.02	0.99–1.04	0.98	0.93–1.04	**1.05**	**1.01–1.09**
HEIGHT (per 1 cm)	1.00	0.98–1.05	1.02	0.94–1.10	1.01	0.97–1.05	1.02	0.98–1.06	1.03	0.92–1.15	1.03	0.98–1.08
Categories of BMI and WC												
≥Overweight (ref: Normal weight)	1.09	0.68–1.75	0.37	0.11–1.31	1.60	0.86–2.98	1.19	0.68–2.07	0.23	0.03–1.72	2.13	0.95–4.76
High WC (ref: Normal WC)	1.08	0.68–1.72	0.45	0.15–1.41	1.48	0.78–2.81	1.28	0.71–2.30	0.23	0.03–1.43	2.16	0.86–5.41
Combined BMI and WC												
Normal weight/Normal WC	Ref.		Ref.		Ref.		Ref.		Ref.		Ref.	
Normal weight/High WC	1.42	0.59–3.45	2.10	0.33–13.15	1.86	0.58–5.93	1.45	0.44–4.74	1.79	0.17–18.29	3.03	0.53–17.46
≥Overweight/Normal WC	1.33	0.65–2.75	1.08	0.20–5.93	2.04	0.75–5.56	1.11	0.47–2.06	1.38	0.17–10.99	2.70	0.73–9.84
≥Overweight/High WC	1.13	0.66–1.92	**0.19**	**0.04–0.98**	1.88	0.90–3.93	1.28	0.67–2.43	0.22	0.03–1.68	**3.28**	**1.10–9.81**

Abbreviations: SD = standard deviation, CI = confidence interval, aOR = odds ratio, BMI = body mass index, WC = waist circumference. Results in bold indicate statistically significant differences (*p* < 0.05). Odds ratios were estimated using conditional logistic regression with controls matched to cases for age, province of residence, educational level, family history of breast cancer, and menopausal status. ^a^ Unadjusted model. ^b^ Adjusted model: adjustments were made for age at menarche (≤12 years or >12 years), nulliparity (yes or no), alcohol consumption (0, 0–12 or ≥12 g/day), and physical activity level (≤8 or >8 MET×h/week). For combined data, BMI was categorized as normal weight (<25 kg/m^2^) and more than overweight (≥25 kg/m^2^) and WC was categorized as normal (≤88 cm) and high (>88 cm).

## Data Availability

The raw data supporting the conclusions of this article will be made available by the authors on request.

## References

[1] International Agency for Research on Cancer Incidencia Del Cáncer En Los Cinco Continentes. https://gco.iarc.fr/en.

[2] ECIS—European Cancer Information System|ECIS—European Cancer Information System. https://ecis.jrc.ec.europa.eu/.

[3] Li X., Yang J., Peng L., Sahin A.A., Huo L., Ward K.C., O’Regan R., Torres M.A., Meisel J.L. (2017). Triple-Negative Breast Cancer Has Worse Overall Survival and Cause-Specific Survival than Non-Triple-Negative Breast Cancer. Breast Cancer Res. Treat..

[4] Asleh K., Riaz N., Nielsen T.O. (2022). Heterogeneity of Triple Negative Breast Cancer: Current Advances in Subtyping and Treatment Implications. J. Exp. Clin. Cancer Res..

[5] Derakhshan F., Reis-Filho J.S. (2022). Pathogenesis of Triple-Negative Breast Cancer. Annu. Rev. Pathol..

[6] American Institute for Cancer Research, World Cancer Research Fund (2018). Diet, Nutrition, Physical Activity and Breast Cancer.

[7] Chen L., Cook L.S., Tang M.T.C., Porter P.L., Hill D.A., Wiggins C.L., Li C.I. (2016). Body Mass Index and Risk of Luminal, HER2-Overexpressing, and Triple Negative Breast Cancer. Breast Cancer Res. Treat..

[8] Aduse-Poku L., Bian J., Gopireddy D.R., Hernandez M., Lall C., Falzarano S.M., Masood S., Jo A., Cheng T.Y.D. (2022). Associations of Computed Tomography Image-Assessed Adiposity and Skeletal Muscles with Triple-Negative Breast Cancer. Cancers.

[9] Taylor C., Sheen M.A., Cattie R., Chung V., Henry M., Olet S., Alberti M., Yuan H., Peeples J., Saxena R. (2023). Obesity and Triple Negative Breast Cancer Diagnosis among Premenopausal Women. J. Clin. Oncol..

[10] Pierobon M., Frankenfeld C.L. (2013). Obesity as a Risk Factor for Triple-Negative Breast Cancers: A Systematic Review and Meta-Analysis. Breast Cancer Res. Treat..

[11] Clinton S.K., Giovannucci E.L., Hursting S.D. (2020). The World Cancer Research Fund/American Institute for Cancer Research Third Expert Report on Diet, Nutrition, Physical Activity, and Cancer: Impact and Future Directions. J. Nutr..

[12] Gómez-Ambrosi J., Silva C., Galofré J.C., Escalada J., Santos S., Millán D., Vila N., Ibãez P., Gil M.J., Valentí V. (2012). Body Mass Index Classification Misses Subjects with Increased Cardiometabolic Risk Factors Related to Elevated Adiposity. Int. J. Obes..

[13] Gómez-Ambrosi J., Silva C., Catalán V., Rodríguez A., Galofré J.C., Escalada J., Valentí V., Rotellar F., Romero S., Ramírez B. (2012). Clinical Usefulness of a New Equation for Estimating Body Fat. Diabetes Care.

[14] MCC-Spain Protocolo de Estudio MCC-Spain Acción Transversal del Cáncer CIBERESP, Versión 16. https://www.mccspain.org/wp-content/uploads/2016/04/01_PROTOCOLO_ESTUDIO_MCC_v16.pdf.

[15] Castaño-Vinyals G., Aragonés N., Pérez-Gómez B., Martín V., Llorca J., Moreno V., Altzibar J.M., Ardanaz E., de Sanjosé S., Jiménez-Moleón J.J. (2015). Corrigendum to: Population Based Multicase-Control Study in Common Tumours in Spain (MCC-Spain): Rationale and Study Design. Gac. Sanit..

[16] World Health Organization (2011). Waist Circumference and Waist-Hip Ratio: Report of a WHO Expert Consultation, Geneva, Switzerland, 8–11 December 2008.

[17] StataCorp (2019). Stata Statistical Software, Release 16.

[18] Torres-De La Roche L.A., Steljes I., Janni W., Friedl T.W.P., De Wilde R.L. (2020). The Association between Obesity and Premenopausal Breast Cancer According to Intrinsic Subtypes—A Systematic Review. Geburtshilfe Frauenheilkd.

[19] Ma L., Liu A., Gao J., Zhao H. (2023). The Prognostic Impact of Body Mass Index on Female Breast Cancer Patients in Underdeveloped Regions of Northern China Differs by Menopause Status and Tumor Molecular Subtype. Open Life Sci..

[20] Gioseffi C., Padilha P.d.C., Chaves G.V., de Oliveira L.C., Peres W.A.F. (2022). Body Weight, Central Adiposity, and Fasting Hyperglycemia Are Associated with Tumor Characteristics in a Brazilian Cohort of Women with Breast Cancer. Nutrients.

[21] Schoemaker M.J., Ellington T., Nichols H.B., Wright L.B., Jones M.E., O’Brien K.M., Weinberg C.R., Adami H.O., Baglietto L., Bertrand K.A. (2025). Central and Peripheral Adiposity and Premenopausal Breast Cancer Risk: A Pooled Analysis of 440,179 Women. Breast Cancer Res..

[22] Bandera E.V., Chandran U., Hong C.C., Troester M.A., Bethea T.N., Adams-Campbell L.L., Haiman C.A., Park S.Y., Olshan A.F., Ambrosone C.B. (2015). Obesity, Body Fat Distribution, and Risk of Breast Cancer Subtypes in African American Women Participating in the AMBER Consortium. Breast Cancer Res. Treat..

[23] Vural A. (2025). Abdominal Obesity and Paraspinal Muscles in Computed Tomography Image: Relationships with Triple Negative Breast Cancer. Am. J. Cancer Res..

[24] His M., Biessy C., Torres-Mejía G., Ángeles-Llerenas A., Alvarado-Cabrero I., Sánchez G.I., Borrero M., Porras C., Rodriguez A.C., Garmendia M.L. (2020). Anthropometry, Body Shape in Early-Life and Risk of Premenopausal Breast Cancer among Latin American Women: Results from the PRECAMA Study. Sci. Rep..

[25] Davis A.A., Kaklamani V.G. (2012). Metabolic Syndrome and Triple-Negative Breast Cancer: A New Paradigm. Int. J. Breast Cancer.

[26] Post L.M., Pathak D.R., Hamilton A.S., Hirko K.A., Houang R.T., Guseman E.H., Sanfelippo D., Carnegie N.B., Olson L.K., Rui H. (2024). Adiposity throughout Adulthood and Risk of Young-Onset Breast Cancer Tumor Subtypes in the Young Women’s Health History Study. Cancer Epidemiol. Biomark. Prev..

[27] Van Mieghem T., Leunen K., Pochet N., De Moor B., De Smet F., Amant F., Berteloot P., Timmerman D., Vanden Bempt I., Drijkoningen R. (2007). Body Mass Index and HER-2 Overexpression in Breast Cancer Patients over 50 Years of Age. Breast Cancer Res. Treat..

[28] Mowad R., Chu Q.D., Li B.D.L., Burton G.V., Ampil F.L., Kim R.H. (2013). Does Obesity Have an Effect on Outcomes in Triple-Negative Breast Cancer?. J. Surg. Res..

[29] Sueta A., Ito H., Islam T., Hosono S., Watanabe M., Hirose K., Fujita T., Yatabe Y., Iwata H., Tajima K. (2012). Differential Impact of Body Mass Index and Its Change on the Risk of Breast Cancer by Molecular Subtype: A Case-Control Study in Japanese Women. Springerplus.

[30] Iyengar N.M., Gucalp A., Dannenberg A.J., Hudis C.A. (2016). Obesity and Cancer Mechanisms: Tumor Microenvironment and Inflammation. J. Clin. Oncol..

